# Improved Enzyme Catalytic Characteristics upon Glutaraldehyde Cross-Linking of Alginate Entrapped Xylanase Isolated from *Aspergillus flavus* MTCC 9390

**DOI:** 10.1155/2015/210784

**Published:** 2015-08-12

**Authors:** Bharat Bhushan, Ajay Pal, Veena Jain

**Affiliations:** ^1^Department of Chemistry & Biochemistry, CCS Haryana Agricultural University, Hisar 125004, India; ^2^HCP Division, ICAR-CIPHET (Central Institute of Post-Harvest Engineering and Technology), Ludhiana, Abohar 152116, India

## Abstract

Purified fungal xylanase was entrapped in alginate beads. Its further cross-linking using glutaraldehyde resulted in large enzyme aggregates which may function as both a catalyst and a support material for numerous substrate molecules. Enzyme cross-linking presented a negative impact on enzyme leaching during repeated washings and recovery of enzyme activity was substantial after twelve cycles of usage. The entrapment followed by cross-linking doubled the total bound activity and also greatly improved the enzyme stability at extreme chemical environment. The wide pH stability, better thermo- and storage stability, lowered *K*_*m*_ value, and protection from some metal ions are salient achievements of present immobilization. The study shows the efficacy, durability, and sustainability of immobilized catalytic system which could be efficiently used for various juice processing operations.

## 1. Introduction

Cloudy fruit juice is a colloidal suspension in which the continuous medium is a solution of pectin, xylan, sugars, and malic acid while the dispersed matter is mainly formed of cellular tissue comminuted during fruit processing [1]. To obtain a clear juice, these suspended particles need to be removed in a process known as clarification. This procedure helps to remove active haze precursors yielding a more clear juice and also decreases the potential for haze formation during storage [2]. Enzymatic hydrolysis using xylanase and associated enzymes plays a very important role in the removal of undesirable turbidity [3].

In view of the variety of their industrial applications and wide range of stabilities under different reaction conditions, xylanases are under extensive study. There has been increasing focus on thermostable acidophilic xylanases in combination with other cell wall degrading enzymes for use in juice clarification and yield improvement [2, 4]. Xylanases improve juice yield by means of liquefaction and hydrolysis of those substances which hinder in juice clarification [2, 5]. However, these enzymes exhibit certain problems such as poor stability, high production costs, and difficulty in recovery. These hurdles should be overcome for worldwide industrial adoption of the enzymes for economic viable process and its commercial realization. Therefore, it is necessary to select the microbial isolate with a potential of higher enzyme production with desirable attributes.

The utilization of free enzymes always presents problems such as low stability, low product recovery, and less repeated use. So the cost-effective immobilization of enzymes for maximum reusability and broader industrial applicability is required. The techniques of macromolecule immobilization have revolutionized the prospects of enzyme applications in industry. Although the best method of immobilization differs from enzyme to enzyme, from application to application, from carrier to carrier, and from peculiarities of specific applications to others, yet the criteria for assessing the robustness of immobilized enzymes remain the same [6, 7].

To date numerous methods of enzyme immobilization are available but the effectiveness of each one depends upon reaction conditions, process of product formation, and its cost evaluation. Different methods, namely, covalent binding, adsorption, adhesion, aggregation, and entrapment, using diverse support like alginate, chitosan, polyacrylamide, agarose, cellulose, dextrans, polymers of polyvinyl alcohol (PVA), and bentonite have been used in the recent past to immobilize either the whole cells or the enzymes. Scientists are actively engaged in manipulating various immobilization methods for various enzymatic transformations and it has been observed that entrapment is the easiest process to scale up because it offers diverse optimization strategies with different procedures and conditions [8].

Entrapment within insoluble calcium alginate beads has been shown to be the most effective approach due to their biocompatibility (nontoxic), low cost, effective particle size, and availability [9–14]. Alginic acid is a polyuronic acid extracted from marine algae and forms gel in the presence of cations such as calcium, zinc, iron, aluminum, and copper [8].

Since xylanase also plays a crucial role in many food processing industries, it was thought worthwhile to immobilize the enzyme on alginate to increase its operational utility on commercial scale. The pores formed with 2% alginate had diameter in the range of 5–200 nm through which small sized enzyme molecules (4–8 nm) can easily leak out. So we tried to cross-link the entrapped enzymes to produce stable enzyme complexes with reduced diffusion characteristics. The present investigation shows the effect of cross-linking modification on the physicokinetic properties of alginate-entrapped enzyme.

## 2. Materials and Methods

### 2.1. Microorganism and Chemicals

*Aspergillus flavus* MTCC 9390 isolated from decaying organic matter in the soil was used in this study. Ammonium sulfate, Sephadex G-100, and oat spelt xylan were products of Sisco Research Laboratory Chemicals (Mumbai, India). Glutaraldehyde solution and sodium alginate were supplied by Fluka (Buch, Switzerland). All other chemicals used were of analytical grade.

### 2.2. Cultivation and Production

The fungal strain (*A. flavus* MTCC 9390) was incubated at 45°C for 144 h in submerged fermentation in preoptimized production medium (pH 6.0) with the following composition (gL^−1^): KH_2_PO_4_, 1.0; NaNO_3_, 2; KCl, 0.5; yeast extract, 0.5; peptone, 0.5; MgSO_4_·7H_2_O, 0.5; FeSO_4_, 0.01; ZnSO_4_, 0.001; CuSO_4_, 0.0005; and oat-spelt xylan, 1.0 as the carbon source [15]. The resulting mycelial mass was separated from broth by filtration through muslin cloth followed by centrifugation at 10000 ×g for 10 min at low temperature to obtain cell-free culture filtrate. The extracellular proteins in supernatant (crude enzyme extract) were precipitated using 70% saturation of ammonium sulphate at 4°C and the obtained precipitates were redissolved in acetate buffer (50 mM, pH 6.0). Xylanase was purified to thirteen folds through molecular exclusion chromatography using Sephadex G-100 [2] and used for immobilization studies.

### 2.3. Optimization of Immobilization

The purified xylanase was immobilized by entrapment within alginate beads. The effects of different concentrations of constituents for building the support material were investigated by varying them to obtain stable alginate-xylanase beads (alginate from 1.0 to 5.0% and CaCl_2_ from 0.05 to 0.3 M). Initially, beads of different sizes were obtained by using needles of variable gauges (G). The beads were collected using filter funnel and stored overnight in 0.02 M CaCl_2_ solution at 4°C for hardening. During entrapment, the ratio of alginate-enzyme solution to calcium chloride was kept 1 : 2 (v/v). The calcium chloride suspension was centrifuged at 3000 ×g for 10 min to estimate unbound enzyme. One gram of beads was treated with 10 mL of glutaraldehyde (1%) prepared in 100 mM MES (2-(N-morpholino)ethanesulfonic acid) buffer (pH 6.0) under continuous magnetic stirring for 90 min at room temperature. At this step, the ratio between beads and glutaraldehyde was opted 1 : 10 as described by us earlier [5]. The treated beads were washed with buffers of 25 mM acetate buffer (pH 5.5) to remove excess glutaraldehyde.

### 2.4. Enzyme Preparations

The purified xylanase was used as free enzyme preparation [2]. The beads prepared without glutaraldehyde treatment were referred to as entrapped enzymes [2] and glutaraldehyde treated beads were referred to as polymer support with cross-linked enzymes.

### 2.5. Enzyme Assay

Twenty-five small beads were suspended in 5 mL of 50 mM acetate buffer (pH 6.0) to calculate the xylanase activity [16]. For the estimation of free and washed out xylanase, one mL of 1% oat spelt xylan solution (in 0.05 M, pH 6.0 acetate buffer) was mixed with 0.1 mL enzyme solution and incubated for 15 min at 60°C. The reaction was stopped by adding 1 mL of 1% 3′,5′-dinitrosalicylic acid (DNS) reagent. The mixture was heated for 5 min at 100°C in a boiling water bath and cooled. Absorbance of sample was measured at 540 nm (*A*_540_) against the substrate blank. A standard curve of xylose ranging from 0 to 1000 *µ*g mL^−1^ was constructed and used to estimate the released xylose. One unit (U) of enzyme activity is defined as the amount of enzyme liberating 1 *µ*mol of xylose equivalents in 1 min under the assay conditions. Protein content was estimated by the method of Lowry et al. [17].

### 2.6. Biochemical and Kinetic Characterization

The effect of temperature and pH on free and bound xylanases (with and without cross-linking) was evaluated in the temperature range 30–90°C and pH range 4.0–7.5. The results of optimal pH determination were obtained by assaying the enzyme preparations at constant substrate (1%) and temperature (60°C). The buffers of constant molarity (100 mM) were acetate (pH 2.0–5.5), MES (pH 5.5–6.5), and glycine–NaOH (pH 7.0–8.5). The pH stability of all enzyme preparations was evaluated by preincubating at different pH values for 60 min at 50°C. Initial activity was considered 100% and the residual activity was measured. Temperature optima were determined by assaying different forms of xylanase at different temperatures. The optimum value of pH was used for different enzyme preparations (i.e., 100 mM MES buffer (pH 5.0 for free, pH 5.5 for entrapped, and pH 6.0 for cross-linked enzyme)). Thermal stability was determined by incubating enzyme preparations at 70°C for 240 min. Enzyme aliquots were withdrawn regularly and the residual activity was calculated. To determine the storage stability, the enzyme preparations were stored at 5 and 25°C. The aliquots were withdrawn at regular intervals and residual activity was determined. The effect of metal ions (5 mM) and chemical inhibitors (1 mM) on xylanase activity was studied at their optimal pH and temperature. Enzyme activity without modifiers was considered 100%. Kinetic constants over a wide range of substrate/xylan concentration (0.5–5.0%) were determined at their optimum pH and temperature. The *K*_*m*_ and *V*_max_ values were calculated from the kinetic data using the double reciprocal plot of Lineweaver and Burk [18]. Reaction progress was monitored by incubating xylan and xylanases for different time intervals (15–60 min) at their optimal reaction conditions.

### 2.7. Desorption and Reusability

Desorption studies were conducted by dropwise pouring the different concentrations of buffered sodium dodecyl sulfate (SDS) on immobilized enzymes. The enzyme activity in bead and washing supernatant was estimated. Enzyme activity in entrapped polymer beads in first run was considered 100%.

### 2.8. Statistical Analysis

The experiments were performed in triplicates and their mean values were taken into consideration for calculation.

## 3. Results and Discussion

### 3.1. Effect of Constituents on Enzyme Immobilization

**Figure 1:**
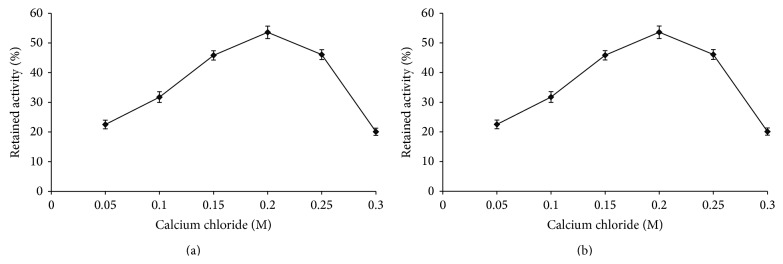
Effect of calcium chloride (a) and duration of cross-linking (b) on percent xylanase activity retention.

Enzyme entrapment in beads depends on the concentration of sodium alginate and calcium ions. So the effect of concentration of these constituents was investigated on immobilization efficiency. Size of the beads also affects the immobilization efficiency. In the present study, xylanase entrapped beads were prepared by passing through a syringe of 18 G needle. The smaller beads obtained with 18 G needles showed highest immobilization efficiency due to increased surface area (data not shown). The immobilization process was further optimized with respect to the concentration of CaCl_2_ (Figure 1). Afterwards, the beads were suspended in glutaraldehyde solution for enzyme cross-linking. The results (Table 1(a)) showed that immobilization efficiency and activity retention increased 16 and 46%, respectively, whereas fold enrichment was enhanced 2-fold by cross-linking. The purpose of adding cross-linker was to cross-link the entrapped enzymes so as to make their aggregates and thereby reduce their leakage. On comparing the unbound and bound activity in the presence and the absence of glutaraldehyde, it was found that cross-linked preparation had minimum leakage (Table 1(a)).

Immobilization of *β*-glucosidase isolated from* Aspergillus niger* in hen egg white was studied by Karimpil et al. [19] who showed that the immobilized enzyme retained approximately 55% activity. Roy et al. [20] reported that xylanolytic activity immobilized on Eudragit L-100 decreased with increase in volume of enzyme loaded on the beads and this was attributed to overcrowding of the enzyme on the surface.

### 3.2. pH Optima and Stability of Entrapped and Cross-Linked Enzyme

**Figure 2:**
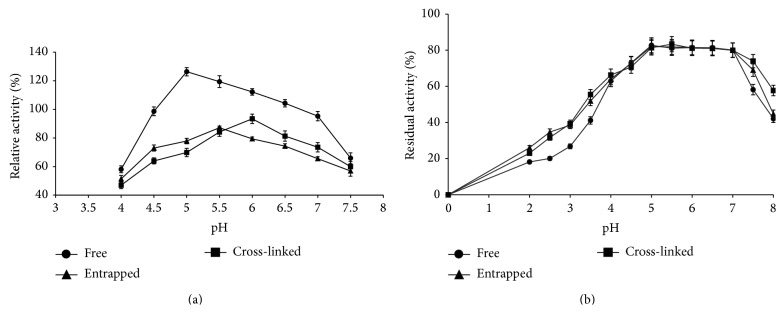
Comparison of pH optima (a) and pH stability (b) of free, entrapped, and cross-linked xylanase from* A. flavus* MTCC 9390.

The pH optima of all the three enzyme preparations, namely, free, entrapped, and cross-linked xylanase, were found in the acidic range. But the pH optima of both the immobilized preparations were on higher side as compared to free enzyme (Figure 2(a)). A slight change in pH optima of cross-linked enzyme was observed which may be due to its surface and residual charge interaction with glutaraldehyde during cross-linking. The pH stability assays showed that preincubation of enzyme preparations in pH range 5.0–7.0 had no negative impact on enzyme activity measured at their optimum pH (Figure 2(b)). Results of the study show that all the three preparations have almost identical acidic pH stability. It was also observed that extreme acidic and alkaline pH were detrimental to protein structure and/or its ionic environment.

Optimum pH of immobilized inulinase was found slightly on lower side (4.0) as compared to its free form (pH 4.5) [21]. Increased pH optima of peroxidase and amylase immobilized in calcium alginate gels and covalently immobilized xylanase have also been documented [5, 13, 22]. A shift in pH optima of pectinase immobilized in alginate using glutaraldehyde was also noticed by Li et al. [23].

### 3.3. Stability Profile of Enzyme Preparations at Different Temperatures

**Figure 3:**
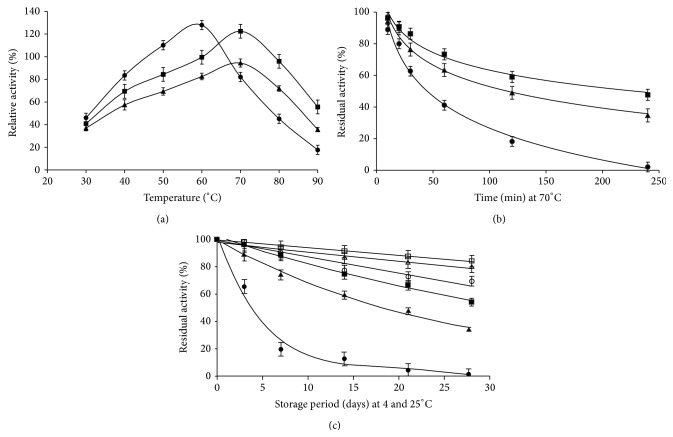
Comparison of temperature optima (a), thermostability (b), and storage stability at 4°C (open legend shapes ○, Δ, and □) and 25°C (closed legend shapes ●, ▲, and ■) (c) of free (●), entrapped (▲), and cross-linked (■) xylanase from* A. flavus* MTCC 9390.

Immobilization with cross-linking increased the thermostability of enzyme without any change in temperature optima as compared to the entrapped enzyme preparation (Figure 3(a)). Study showed that cross-linked enzyme retained 48% activity after 240 min at 70°C which was higher than the other two preparations (Figure 3(b)).

Storage stability studies showed that cross-linked enzyme preparation retained 54% activity as compared to 32% of entrapped preparation after 28 d storage at 25°C (Figure 3(c)). This enhanced storage stability could be due to the better physical contacts or structural rigidness or stabilization of the enzyme. Similar results after cross-linking have been documented by Nwagu et al. [22]. The improved thermal stability at 60°C was obtained for lipase immobilized on chitosan-alginate beads activated with 2% glutaraldehyde. The immobilized enzyme was 33 times more thermostable than the soluble enzyme [12]. Thermostability studies of immobilized pectinase revealed that it retained >80% of its initial activity after 5 d storage at 30°C while its free form retained only 30% of initial activity [24]. Coimmobilization in the alginate fibers and beads also resulted in protein leaching and decreased enzymatic activity after one month storage at 4°C [10]. Barbosa et al. [25] opined that thermal stabilizations of lipase-B at different pH values are consequences of both intramolecular chemical modification and intermolecular cross-linking which create the least damaging local environment.

Two independent variables, namely, pH and temperature optima, of xylanase catalyzed reaction were directionally displaced to different extent depending upon the nature of enzyme preparation (free or bound) and this shift is inherent feature of immobilization process [2, 5, 23]. In our previous study on thermoinactivation rate analysis of fungal xylanase, we found that thermal denaturation does not lead to unfolding but aggregation [26].

### 3.4. Kinetic Constants and Velocity Saturations of Enzyme Preparations

**Figure 4:**
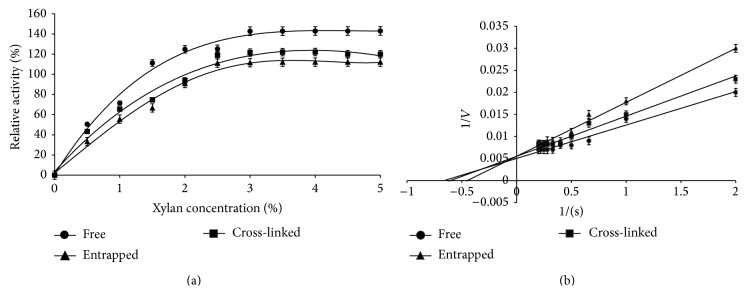
Comparison of saturation velocity curve (a) and double reciprocal plot (b) of free, entrapped, and cross-linked xylanase from* A. flavus* MTCC 9390.

Immobilized enzymes are generally surrounded by a substrate concentration that is lower than the bulk concentration. Two different immobilization effectiveness factors, namely, stationary (*η*) and operational (*η*_0_), were determined from substrate saturation curve and reaction progress curve, respectively (data not shown). Kinetic behavior of the free, entrapped, and cross-linked enzyme preparations showed a typical rectangular hyperbolic response with increasing concentration of substrate, a characteristic of Michaelis-Menten kinetics (Figure 4(a)). Free enzyme had *K*_*m*_ and *V*_max_ of 1.53% and 200 U mL^−1^, respectively, while a small decrease in *K*_*m*_ (1.47%) and *V*_max_ (187 U mL^−1^) of cross-linked enzyme preparation was observed (Figure 4(b)). It might be caused by either external (*η*_*e*_) or internal (*η*_*i*_) stationary effectiveness factors which correspond to diffusion layer and stearic hindrance inside the particles, respectively.

The apparent *K*_*m*_ of lactase immobilized in carboxymethyl cellulose-alginate beads was 107.24 mM as compared to 95.57 mM for entrapped lactase in alginate. It might be due to increased surface area of beads and least diffusion resistance faced by substrate to accumulate inside the beads [27]. The apparent *K*_*m*_ (2.08 mM) and apparent *V*_max_ (0.95 *µ*mol min^−1^) of immobilized lipase were lower as compared to free lipase (*K*_*m*_ 8.0 mM; *V*_max_ 2.85 *µ*mol min^−1^) [19]. Similar work suggests that alginate network limits the permeation rates of substrate and product. Contrarily, polyacrylamide-entrapped peroxidase showed relatively lower *K*_*m*_ value as compared to native peroxidase [28]. Complexion of amylase and glucosidase in alginate with pullulan not only decreased the *K*_*m*_ but also increased the *V*_max_ [29].

### 3.5. Impact Assessment of Metal Ions and Chemicals

Studies on the effect of metal ions and modifiers suggested that immobilization by cross-linking method had a protective effect against harmful chemicals like mercury chloride and lead acetate commonly used in industrial processes (Table 1(b)). Interestingly, the free enzyme was activated by sodium chloride and potassium chloride whereas both the immobilized preparations were inhibited to varying extent. The reason behind this could not be ascertained in our study. The partial restoration of enzyme activity is in agreement with the previous report [30].

### 3.6. Reaction Progress

**Figure 5:**
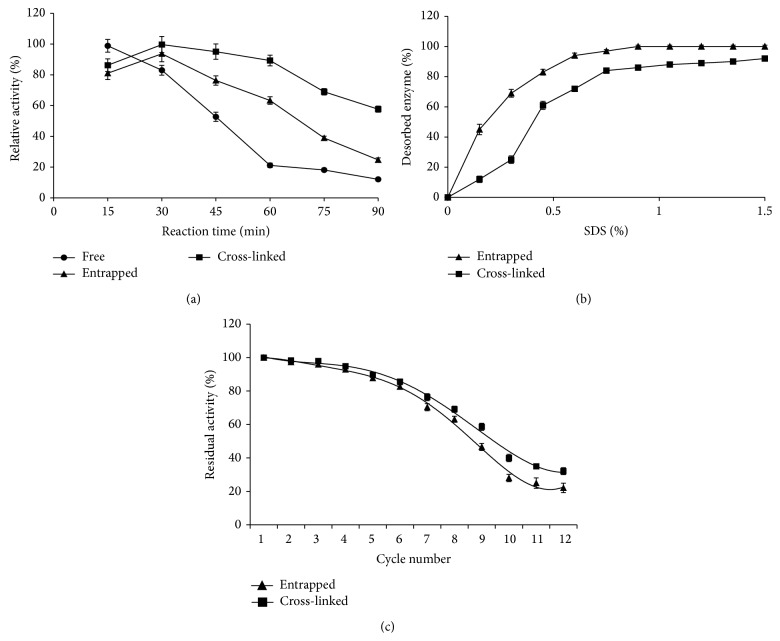
Reaction progress curve (a), desorptivity (b), and reuse of cross-linked and entrapped enzyme preparation (c) from* A. flavus* MTCC 9390.

It was observed that, after entrapment and cross-linking, the reaction retention time for maximal activity was increased to 30 min from 15 min of free enzyme (Figure 5(a)). The observed increase in reaction time is due to diffusion of substrate molecules into calcium alginate beads which require greater time to reach the substrate binding site of immobilized enzyme.

As the reaction progresses, the substrate concentration outside the beads and stationary effectiveness factor get changed. So the operational effectiveness factor comes into action which is clear from increased reaction time in both the immobilized enzyme preparations in comparison with free enzyme. Increased reaction time indicates increased resistance to diffusion faced by high molecular weight substrate. Similar results have been presented with microbial pectinase where time increased from 5 to 10 min after immobilization [24, 31].

### 3.7. Reusability

The most important parameter of immobilized enzymes for industrial application is their repeated use. So the operational stability of xylanase entrapped in alginate gel was assessed by reusing the immobilized enzyme for twelve cycles (Figure 5(c)). It was observed that, up to four cycles, there was no appreciable loss in activity but afterwards the activity started to decrease. After eight cycles, 63% of the initial activity was retained by entrapped enzyme. This loss in activity was concomitant with the reduction in protein content in beads (data not given). The immobilized xylanase was found to have good operational utility as evident by substantial retention of activity after eight consecutive cycles. After twelve cycles, most of the enzyme leaked out and the difference in activity in two preparations was ~10%. The loss in activity upon reusing of immobilized preparation is a general observation. A lesser loss in activity in cross-linked enzyme preparation may be attributed to the cross-linking of enzyme molecules by glutaraldehyde.

Significant loss in activity of immobilized xylanases has been reported by many workers [2, 24, 32]. They have shown that xylanases from various sources immobilized on various support could be reused up to six to ten cycles. The operational stability of enzymes has been reported to increase by immobilization and suggested to be exploited on industrial scale. Alginate entrapped lipase was active after 10 cycles without any loss in activity [33]. However, 40 to 50% loss in activities of xylanase and lipase entrapped on *κ*-carrageenan and calcium alginate beads has also been observed [34, 35]. No significant change in xylan hydrolysis by immobilized preparation up to ten cycles was observed by Roy et al. [20], while reusability of up to eight cycles has been reported by Silva et al. [12].

### 3.8. Desorptivity

It is important to note that, after a number of reuse cycles, enzyme gets deactivated and it should be removed from the matrix. The studies performed on desorption were also meant to take into consideration the marginal amount of enzyme likely to be loosely entrapped. It was found that 0.5% SDS was enough to desorb the enzyme from matrix even after cross-linking with glutaraldehyde (Figure 5(b)). This might be due to the competition of anionic surfactant with enzyme surface bound to the matrix.

Our results are in agreement with earlier reports which also provided similar suggestions [25, 36]. Researchers have always paid attention to strongly cross-link the enzyme molecules in the polymer network with the help of linkers, aggregators, carriers, and composite materials [13, 27, 29, 37].

## 4. Conclusion

The application of xylanase in fruit juice industry includes clarification, liquefaction, and maceration of fruit pulp to increase juice yield. So the enzymes which have desirable characteristics such as the maximum reusable activity at high temperature and acidic pH have great potential since they can be introduced in different processing industries. Based on the observations, it could be suggested that* A. flavus* MTCC 9390 can be exploited at industrial scale for the production of xylanase. Immobilization with the cross-linking method improved the desirable characteristics such as thermostability and operational utility of the enzyme. So this method can be used to prepare xylanase-alginate beads for better xylanolytic activity and stability. Considering the method of enzyme employment and its effective reusability, this piece of work may contribute to pilot plant development of immobilized xylanase. But its commercial potential is still to evaluate since other studies like bioreactor output and food quality are equally essential.

## Figures and Tables

**(a):** 

Preparations	Theoretical maximal bound activity (U)	Unbound activity (U)	Bound (actual) activity (U)	Immobilization efficiency^*∗*^	Activity retention^#^	Fold enrichment^†^
Entrapped	114.67 ± 4.15	98.55 ± 3.06	87.4 ± 3.63	76 ± 2.24	53 ± 3.55	41 ± 3.00
Cross-linked	210.14 ± 6.31	3.08 ± 0.35	194.75 ± 6.53	92 ± 3.17	99 ± 3.78	91 ± 7.00

^*∗*^Immobilization efficiency = (actual bound activity/maximum theoretical bound activity) × 100.

^#^Activity retention = (maximum theoretical bound activity/total activity employed for immobilization) × 100.

^†^Fold enrichment = (actual bound activity/total activity employed for immobilization) × 100.

Note: immobilization effectiveness factor can be calculated from immobilization efficiency by dividing it with 100. Immobilization yield can be calculated from fold enrichment by dividing it with 100.

Enzyme units used for the immobilization: 213.22 UmL^−1^.

**(b):** 

	Residual activity (%)		
	Free enzyme	Entrapped enzyme	Cross-linked
Metal ions (5 mM)			
NaCl	124.9 ± 5.66	73.6 ± 2.92	74.2 ± 3.21
KCl	166.6 ± 6.84	92.1 ± 4.25	96.9 ± 4.47
MgCl_2_	93.8 ± 3.62	57.5 ± 2.43	63.5 ± 3.02
CaCl_2_	84.6 ± 2.68	87.6 ± 3.73	80.6 ± 3.45
CoCl_2_	33.4 ± 1.32	41.1 ± 1.64	38.0 ± 1.12
HgCl_2_	9.8 ± 1.02	37.04 ± 2.46	39.9 ± 1.82
CdCl_2_	15.9 ± 1.68	36.8 ± 2.37	35.9 ± 2.54
NiCl_2_	59.9 ± 5.23	47.4 ± 4.02	49.8 ± 4.17
Lead acetate	35.2 ± 2.63	73.2 ± 3.61	78.9 ± 4.23
Chemicals (1 mM)			
PMSF^*∗*^	97.3 ± 5.62	104.9 ± 6.10	127.3 ± 6.55
PHMB^#^	4.7 ± 0.58	34.7 ± 2.81	47.6 ± 3.19
DTNB^†^	5.0 ± 0.95	29.4 ± 1.66	53.2 ± 2.58
EDTA^‡^	90.8 ± 6.24	82.71 ± 5.77	84.5 ± 5.29

^*∗*^PMSF: phenylmethanesulfonyl fluoride.

^#^PHMB: para-hydroxymercurybenzoate.

^†^DTNB: 5,5′-dithiobis-(2-nitrobenzoic acid).

^‡^EDTA: ethylenediaminetetraacetic acid.
